# Performance of a Personalized Smart Cueing Device to Detect Freezing of Gait in Parkinson's Disease

**DOI:** 10.1111/ejn.70589

**Published:** 2026-07-09

**Authors:** Matthijs van der Laan, Erwin E. H. van Wegen, Jens A. N. Keijser, Sander A. L. M. Minnoye, Rein van Zutven, Vincent de Groot, Marc B. Rietberg, Jorik Nonnekes

**Affiliations:** ^1^ Department of Rehabilitation Medicine Amsterdam UMC Amsterdam the Netherlands; ^2^ Amsterdam Movement Sciences Rehabilitation & Development Amsterdam the Netherlands; ^3^ Cue2Walk International B.V The Hague the Netherlands; ^4^ Donders Institute for Brain, Cognition and Behaviour; Department of Rehabilitation; Centre of Expertise for Parkinson & Movement Disorders Radboud University Medical Centre Nijmegen the Netherlands

**Keywords:** freezing of gait detection, latency, Parkinson's disease, sensitivity, specificity

## Abstract

External cueing is a proven strategy to overcome freezing of gait (FoG) in Parkinson's disease (PD). However, manual activation of cueing is often too late and continuous cueing may be perceived as burdensome. Therefore, the Cue2walk, a medical device for FoG detection and provision of external cues, was developed. This study aimed to determine (1) the performance to detect FoG of the Cue2walk device and (2) the effect of personal optimization on its performance to detect FoG. Twenty‐four people with PD and daily FoG completed a gait circuit including FoG‐triggering tasks in their own home environment, while wearing a Cue2walk device to collect acceleration data. Two experienced independent raters rated the occurrence of FoG using video annotation. The data set of each participant was split into a training (60%) and test set (40%), obtaining an even distribution of FoG episodes. The training set was used to personalize the settings of the algorithm. The test set was subsequently used to assess the performance to detect FoG of the standard and personalized setting by calculating sensitivity, specificity, and latency (time between onset and detection of FoG). Sensitivity was 28.2% ± 31.6%, specificity was 96.0% ± 4.1%, and latency was 1.9 s ± 2.4 s on the test set with the standard settings. With personalized settings, sensitivity was 89.1% ± 14.8%, specificity was 93.3% ± 5.5%, and latency was 1.6 s ± 2.2 s. Sensitivity was significantly higher for the personalized settings (*p* < 0.001), while latency and specificity remained similar. The Cue2walk device showed good performance to detect FoG when using the personalized settings. The clinical effectiveness needs to be determined in future research.

## Graphical abstract

External cueing can help overcome freezing of gait (FoG) episodes. The Cue2walk device delivers cueing signals when FoG is detected, but its detection performance was previously unknown. This study, conducted in a home‐based environment, showed that the standard settings of the device result in poor FoG detection performance, whereas the personalized settings substantially improved performance and remained robust on unseen data.

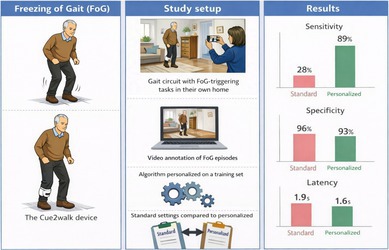

**Abbreviations**
FN: false negative
FoG: freezing of gait
FP: false positive
MDS‐UPDRS: Movement Disorders Society Unified Parkinson's Disease Rating Scale
MMSE: Mini‐Mental State Examination
NFOG‐Q: New Freezing of Gait Questionnaire
PD: Parkinson's disease
ROC: receiver operating characteristic
TN: true negative
TP: true positive

## 1 Introduction

Freezing of gait (FoG) affects more than 50% of the total population of people with Parkinson's disease (PD) (Zhang et al. 2021). It is clinically defined as “paroxysmal episodes wherein there is an inability to step effectively, despite attempting to do so” (Gilat et al. 2026). FoG increases the risk of falls (Latt et al. 2009), results in fear of falling (Bloem et al. 2004; Grimbergen et al. 2013), and a subsequent loss of mobility (Bloem et al. 2004; Moore et al. 2007; Rider et al. 2025). As such, it is one of the most disabling motor symptoms of PD and severely affects quality of life (Cronin et al. 2024).

FoG is not fully relieved by pharmacological and/or surgical interventions (Gámez‐Leyva and Cubo 2024; Molina et al. 2021), so complementary nonpharmacological interventions are needed (Tosserams et al. 2025). These include the use of compensation strategies, such as external rhythmic cueing. External cueing is defined as “using external temporal or spatial stimuli to facilitate movement (gait) initiation and continuation” (Nieuwboer et al. 2007). Such stimuli can be auditory, visual, or somatosensory (Nonnekes et al. 2019). Several devices exist that integrate a form of external cueing (Ahn et al. 2017; Dvorani et al. 2024; Porciuncula et al. 2025; Zoetewei et al. 2024). Typically, external cueing is manually triggered (e.g., by switching on a metronome) or continuously present (e.g., stripes on the floor). However, since manual triggering of cues is often too late and lack of initiative is a common symptom in PD (Poewe 2008), it has limited effectiveness during a sudden FoG episode. Continuous cueing, on the other hand, may be experienced as burdensome and intrusive.

These limitations can be addressed by smart cueing. Smart cueing means that external cueing will be activated automatically upon detection of a FoG episode by a wearable device. In this way, cueing will only be activated when needed, i.e., when a FoG episode is present, without the need for manual activation. For smart cueing, it is key that the device correctly identifies FoG episodes. Additionally, the time between the onset and the detection of a FoG episode (latency) needs to be adequate for the cue to be helpful for the person with PD to overcome a FoG episode.

Two recent reviews demonstrated that various studies have shown good performance to detect FoG using a variety of device set‐ups (Elbatanouny et al. 2024; Zhang et al. 2024). However, these set‐ups often used multiple sensors, which limit practicality and usability during everyday life. More importantly, most of these device set‐ups do not offer cueing upon detection (yet). The Cue2walk CE class 1 medical device (Cue2Walk International B.V., The Hague, The Netherlands) addresses these limitations. It is a small, single‐sensor smart cueing device, which is placed just below the lateral side of the knee on the leg that is most affected by FoG using a Velcro strap. The device is equipped with an accelerometer that measures accelerations in three directions in a local reference frame with a nominal sample frequency of 32.33 Hz. FoG episodes are detected based on real‐time acceleration data. The detection algorithm is two‐layered, i.e., the device first determines whether the user is moving or not. If so, the acceleration data are processed, using up to six algorithms, with each algorithm having a similar weight factor. The algorithms are based on, among others, the Freeze Index (Moore et al. 2008) and are partly time‐based and partly frequency‐based. More information on the device and the algorithm is provided in the patents published by the manufacturers of the device (Minnoye et al. 2023).

To address heterogeneous manifestations of FoG episodes (i.e., there is large variation in the kinematic characteristics of FoG between people with PD), the detection algorithm of the Cue2walk device can be personally optimized by determining the set of active algorithms and the thresholds of the active algorithms for each user individually. If a personally predetermined set of the six algorithms signals a FoG episode, the Cue2walk device will detect a FoG episode and subsequently generate auditory and/or vibratory cues. The Cue2walk device also offers a standard setting for the detection of FoG, which is based on the average of the personally optimized settings of 50 users of the Cue2walk device. In addition to that, the user of the device has four sensitivity options around both the standard and personally optimized setting: more, much more, less, and much less sensitive to detect FoG than the standard or personally optimized setting.

Previous research suggests the Cue2walk device could be a valuable tool for managing FoG (van der Laan et al. 2025). However, its performance to detect FoG is unknown. Therefore, the aim of this study was to determine the sensitivity, specificity, and latency of the Cue2walk device, and to determine the effect of personal optimization of its detection algorithm on these performance metrics. Although the Cue2walk device offers smart cueing capabilities, the assessment of its therapeutic efficacy and comparisons with other cueing devices were beyond the scope of the present work.

## 2 Materials and Methods

### 2.1 Participants

We recruited 24 people with PD who are experiencing daily FoG. This sample size is in accordance with sample sizes used in previous literature (Elbatanouny et al. 2024). The eligibility criteria were idiopathic PD (defined by UK Brain Bank Criteria), diagnosed by a neurologist; disabling/regular presence of FoG (defined as a score of 4 [“Very often, more than one time a day”] on Question 2 [“How often do you experience FoG?”] from the New Freezing of Gait Questionnaire [NFOG‐Q] [Nieuwboer et al. 2009]); ability to communicate with the investigator and follow instructions sufficiently; and ability to walk for at least 3 min consecutively with cane or walker if needed. The exclusion criteria were neurological comorbidities or orthopedic conditions hampering mobility (e.g., wheelchair‐dependent, severe arthrosis or neuropathy). The study was approved by the Medical Ethical Committee Arnhem‐Nijmegen (reference number 2023‐16413). Each participant gave written informed consent prior to entering the study.

### 2.2 Measurement Protocol

**FIGURE 1:**
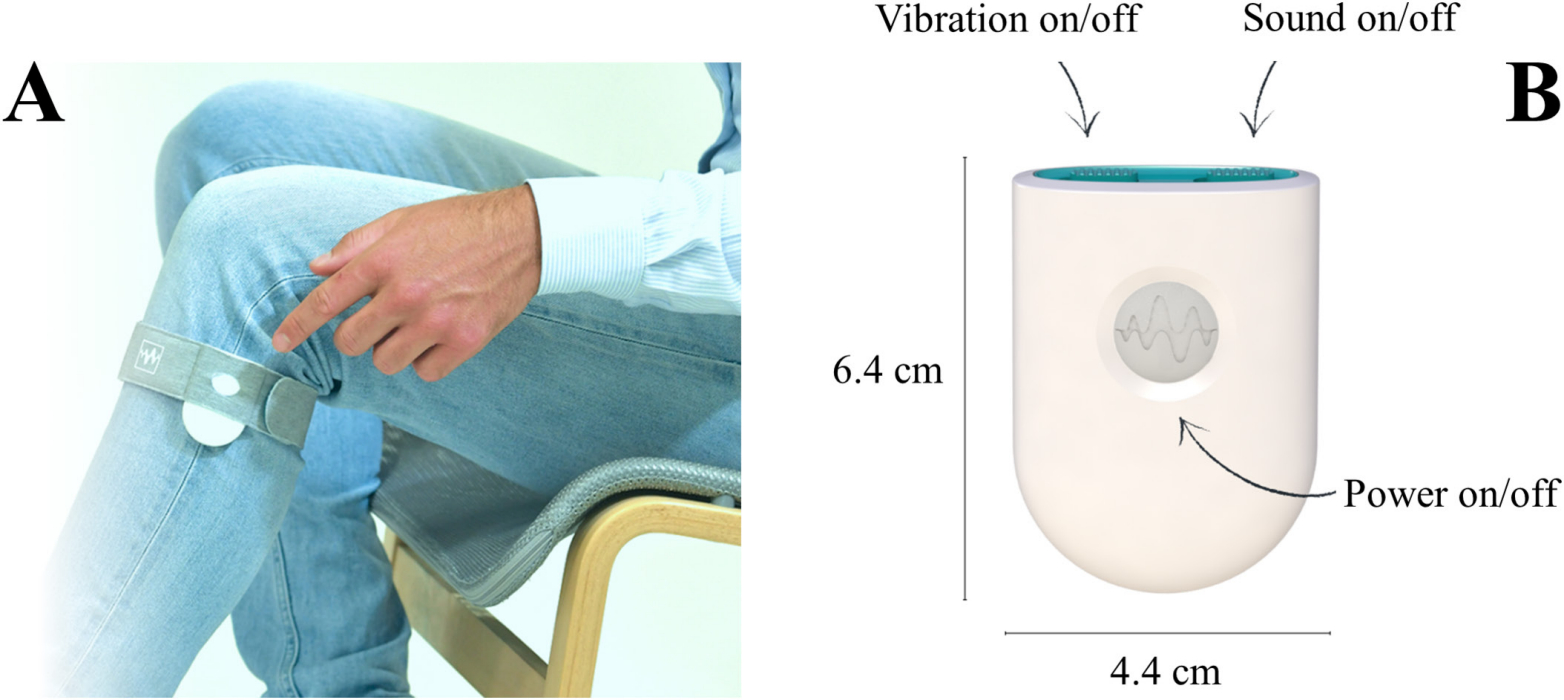
Images of the Cue2walk device. (A) The Cue2walk device attached to the lower leg. (B) The dimensions of the Cue2walk device and the function of the button and the two sliders on the device.

**FIGURE 2:**
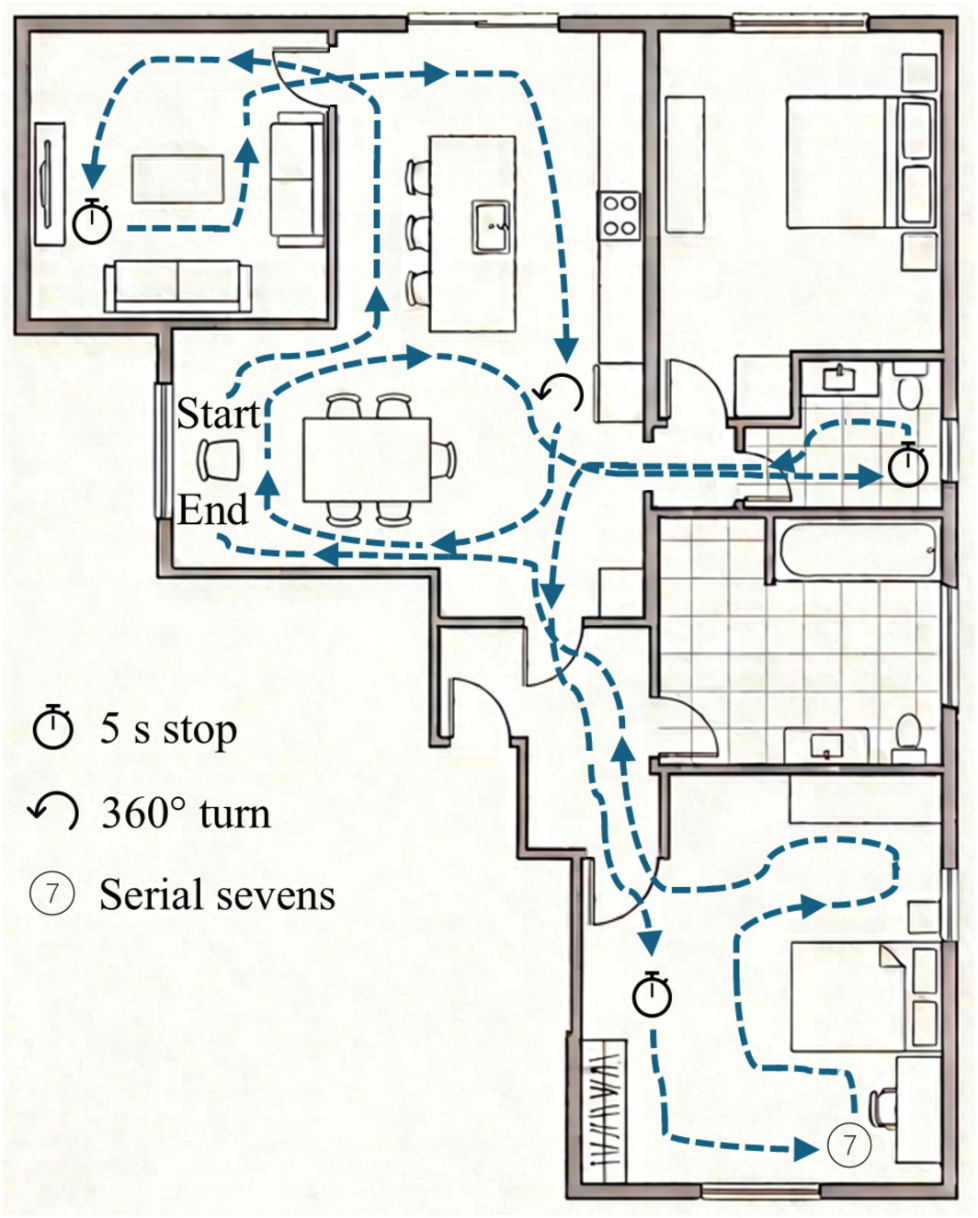
Visual example of a gait circuit, including freezing of gait‐triggering tasks, at the home of a participant.

During an intake before each visit, we asked the participants whether FoG occurred during the ON‐medication phase. If so, the measurements were conducted in an ON‐medication phase (< 1 h after dopaminergic medication intake). If participants indicated that FoG only occurred during the OFF‐medication phase, the measurements were conducted in an OFF‐medication phase (> 12 h after medication intake). During the intake, we also asked participants to answer Question 2 (“How often do you experience FoG?”) of the NFOG‐Q, to verify regular presence of FoG. Important to note is that we did not ask participants to complete the entire NFOG‐Q. The measurements were conducted at the house of the participant. First, the assessor asked the participant for spots in the house that would generally trigger FoG, such as narrow spaces and doorways (Lewis and Shine 2016). In addition to the personal FoG‐triggering spots, the gait circuit consisted of a number of standard walking tasks that were performed by all participants, such as walking around furniture and walking between the table and a chair. Thereafter, the assessor attached the Cue2walk device (Figure 1) to the most affected leg of the participant and asked the participant to start walking, from a sitting position in a chair, at comfortable walking speed. All cueing functionalities were turned off, so the device was used only as a sensor to collect data. The measurement was recorded on video with an iPhone XR (Apple Inc., Cupertino, CA, USA) with a frame rate of 30 fps. At the start of the measurement, the central button on the Cue2walk device was shortly pressed by the assessor to create a synchronization point for the video and the acceleration data. The time point at which the central button is pressed is saved as an event in the data collected by the Cue2walk device, and it can be seen on the video since the central button will light up. Hereafter, the assessor guided the participant through the predetermined personal FoG‐triggering spots and the standard walking tasks. Besides walking, the participant was asked to stand still for at least 5 s on three separate occasions. A visual example of a gait circuit at the home of a participant is depicted in Figure 2.

The aim of the measurement was that the participant displayed a minimum of five FoG episodes during the gait circuit through the house. This minimum number was pragmatically selected to ensure reliable evaluation of the device's FoG detection performance while avoiding excessive participant burden. Double tasks, such as serial subtraction (serial sevens) and spelling words backwards, were given if a FoG episode did not occur within the first 2 min of the measurement. If after the minimum duration of 3 min the desired number of FoG episodes was reached, the assessor led the participant back to the chair where the measurement started, after which the measurement was completed. Otherwise, the measurement was extended to a maximum of 8 min. If during this extended period the desired minimum number of FoG episodes was reached, the assessor led the participant back to the chair, and the measurement was completed. Otherwise, if the minimum number of FoG episodes was not reached during the extended period, the measurement was ended after 8 min. The measurement was also ended when the participant did not display FoG episodes for 3 min after the initial minimum of 3 min, or when the participant indicated to be too fatigued to continue.

After the measurement, two additional examinations were performed: The severity of motor symptoms was assessed by the Movement Disorders Society Unified Parkinson's Disease Rating Scale (MDS‐UPDRS) part III (Goetz et al. 2008), and cognitive function was assessed using the Mini‐Mental State Examination (MMSE) (Folstein et al. 1975).

### 2.3 FoG Video Annotation

The measurements were video‐recorded to be able to annotate after the measurement when FoG episodes occurred. The video annotation was performed independently by two trained raters to mark the FoG episodes more accurately (Cockx et al. 2022). Open‐source video processing program ELAN (version 6.8) was used for the annotation of the FoG episodes, and Kinovea (version 0.9.5) was used to obtain the sample numbers of the annotated FoG episodes. The onset of an FoG episode was defined as “the first moment that any part of the foot is lifted off the ground as part of the first ineffective step or the first moment when a visible, self‐reported or objectively documented attempt to take a step is noted” and the end of a FoG episode was defined as “the first moment that any part of the foot is lifted off the ground as part of the first of two steps that resemble typical or quasi‐normal stepping performance in the same individual under similar task conditions or when there is no longer a visible, self‐reported or objectively documented attempt to take a step” (Gilat et al. 2026). Discrepancies between raters were discussed until consensus was reached (Cockx et al. 2022). FoG episodes shorter than 1 s were not included in the analysis, since, in the case of such short episodes, the demand for cueing is often already over before the FoG episode is detected. Furthermore, if the device does detect the short FoG episode, only one cueing signal will be given during the episode, and the rest of the signals will be given when the episode is not present anymore, which might be experienced as burdensome and could reduce the utility of the cueing signals.

### 2.4 Data Analysis

The acceleration data, keyframes, and video details were loaded into MATLAB (The Mathworks, Natick, MA, USA), version R2023b. The keyframes in which a FoG episode occurred were synchronized to the acceleration data collected by the Cue2walk device. Since the frame rate of the video (30 fps) and the sample frequency of the acceleration data (32.33 Hz) differed, the keyframes were multiplied by 32.33/30 to obtain the corresponding samples in the acceleration data. Subsequently, the acceleration data were run through the Cue2walk algorithm, using the standard setting and the personally optimized setting. The algorithms assessed a 1‐s sliding window, which shifts one sample per iteration.

The interrater agreement was calculated using Cohen's Kappa, positive agreement, and negative agreement (Byrt et al. 1993; Cicchetti and Feinstein 1990; Cockx et al. 2022). Cohen's Kappa underestimates the level of agreement when prevalence of events is low (i.e., high prevalence index) and overestimates the level of agreement when bias of the raters is high (i.e., high bias index) (Byrt et al. 1993; Cicchetti and Feinstein 1990; Cockx et al. 2022; Feinstein and Cicchetti 1990; Sim and Wright 2005). Therefore, the prevalence index and bias index were also calculated and presented alongside Cohen's Kappa. For Cohen's Kappa, positive agreement and negative agreement, values of < 0.00, 0.00–0.20, 0.21–0.40, 0.41–0.60, 0.61–0.80, and 0.81–1.00 were considered poor, slight, fair, moderate, substantial, and almost perfect, respectively (Cockx et al. 2022; Landis and Koch 1977).

Each measurement was split into a training set and a test set. We investigated multiple time ratios (40/60, 50/50, 60/40, and 70/30) to determine which ratio resulted in an even distribution of FoG episodes between the training set and test set. The 60/40 ratio resulted in the most even distribution and was therefore used in further analysis. In the first 60% of each measurement, the set of active algorithms and the thresholds of the active algorithms were determined for each participant individually. In the last 40% of each measurement, the performance of the Cue2walk device to detect FoG was determined for each participant, using the algorithm settings as determined in the training set of that measurement. If during a measurement the onset of a FoG episode was at the end of the 60% of the measurement and lasted through the beginning of the last 40% of the measurement, the FoG episode was included in the training set. The test set of that measurement would then start the first sample after the end of the FoG episode.

**FIGURE 3:**
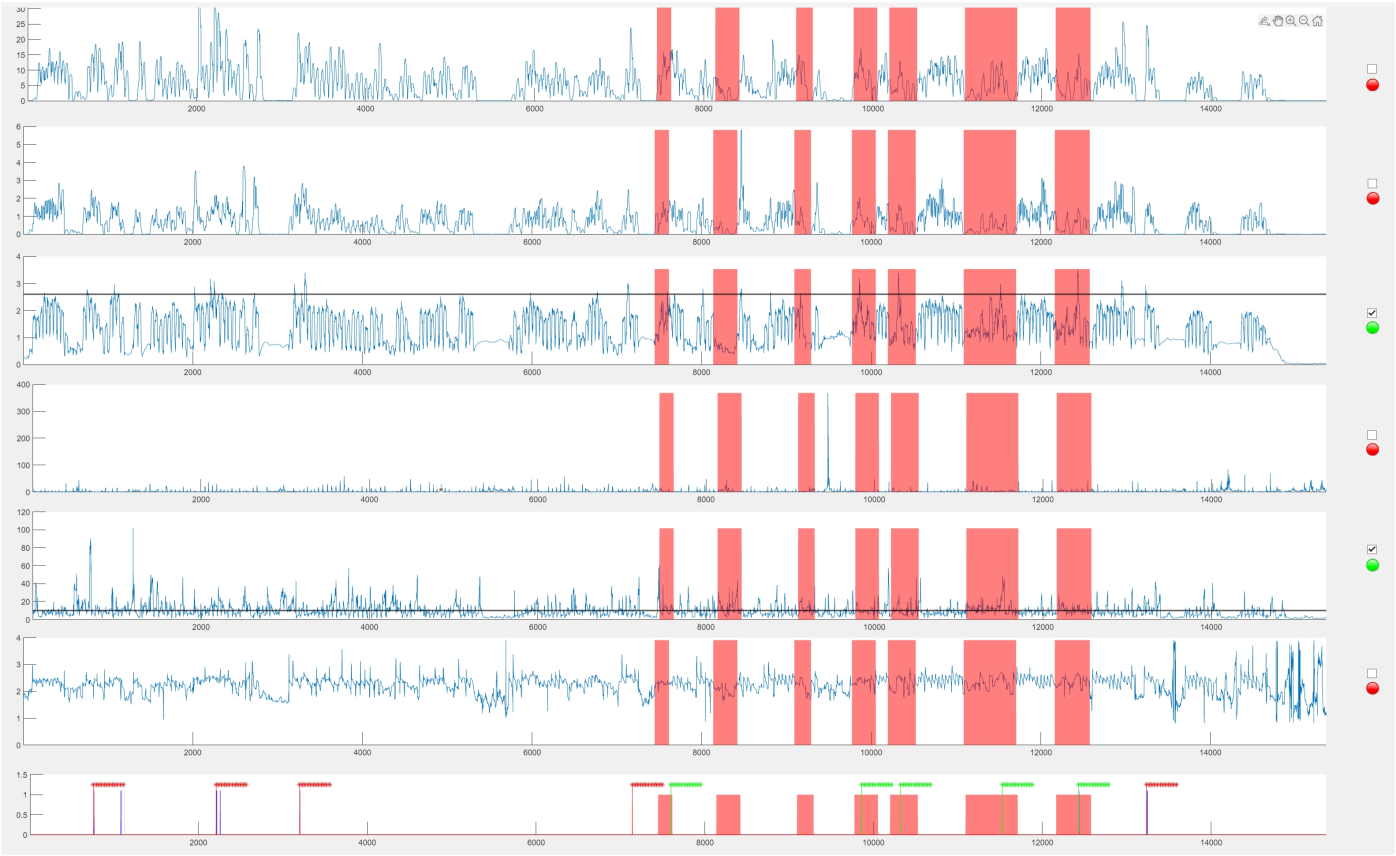
Typical example of the Cue2walk algorithm when acceleration data are loaded into MATLAB. The red squares indicate where freezing of gait (FoG) episodes occurred as determined by video annotation. The upper six plots display the output when the acceleration data are processed by the six different algorithms. The third and fifth algorithms are selected for this participant (as can be seen by the checked boxes and green dots on the right), since they result in the highest true positive and the lowest false positive events. The lower plot displays the final result. The green vertical lines (inside the red squares) indicate the true positive events, whereas the red vertical lines (outside the red squares) indicate the false positive events. The blue vertical lines also indicate the detection of a FoG episode, but since they occur within the duration of a previously triggered cue, these detections will not trigger another cue. In this example, five out of seven FoG episodes were correctly detected, whereas the algorithm produced five false positive events.

In this study, the performance to detect FoG of the standard setting and the personally optimized setting of the Cue2walk device were determined. For the personally optimized setting, the thresholds were chosen after collecting the data and were determined based on their capability of detecting FoG episodes in the training set of the measurement. The training set and test set of each measurement were stored in a separate file. Therefore, during personal optimization of the settings, the operator was blinded to the test set of that measurement. Finding the optimal thresholds was done manually by an experienced operator via trial and error using the tool shown in Figure 3. This tool matches the on‐device real‐time behavior. In an iterative process, the set of algorithms and their corresponding thresholds were adjusted to identify a combination that maximized true positive (TP) events and minimized false positive (FP) events. This process was guided by stepwise evaluation of TP and FP events after each adjustment rather than by an exhaustive or formal optimization of the parameter space. Convergence toward a final set of parameters was achieved heuristically, based on the operator's expertise and prior experience with the personalization tool. This experience enabled the operator to focus on a subset of plausible parameter regions and to disregard combinations that led to clearly suboptimal performance early in the process. Of the six algorithms, the first two were selected most frequently, whereas the sixth was selected the least. If a participant did not display FoG in the training set of the measurement, the thresholds of the algorithms were placed directly above the highest peak of the acceleration data.

The performance of the Cue2walk device was quantified in terms of sensitivity, specificity, and latency. The following formula was used to calculate sensitivity:

$$\text{sensitivity}\;\left(\%\right)=\frac{\mathrm{TP}}{\mathrm{TP}+\mathrm{FN}}\times 100$$

where TP is the number of true positive events and FN is the number of false negative events.

If the algorithm is triggered multiple times during a single FoG episode, this will be counted as one TP event.

The number of times no FoG episode occurred and the Cue2walk device correctly did not detect one (TN) cannot be expressed in absolute numbers. Therefore, we divided the parts of the measurement in which the participant did not display FoG and was not standing still into bins of 1.5 s. The bin length was based on the average cadence (80–90 steps per minute) collected in > 100 users of the Cue2walk device and allows for two steps to be taken by the participant in every bin. Subsequently, we determined the number of bins the Cue2walk device falsely detected a FoG episode (FP) for the standard and personally optimized setting. The remaining bins are TN bins. The following formula was used to calculate specificity:

$$\text{specificity}\;\left(\%\right)=\frac{\mathrm{TN}}{\mathrm{FP}+\mathrm{TN}}\times 100$$

where TN is the number of true negative bins and FP is the number of false positive bins.

If FPs occur within five bins of each other, they will be merged together.

First, sensitivity and specificity were calculated per participant. Hereafter, these individual values were averaged to determine the sensitivity and specificity of the Cue2walk device. Because sensitivity and specificity were computed at different levels of analysis (i.e., event‐based vs. time‐based), the components of the confusion matrix could not be defined on a common set of observations. Consequently, receiver operating characteristic (ROC) analysis was not appropriate, and an ROC curve was not included.

Latency was defined as the time between the onset of a FoG episode, as determined during video annotation, and the detection of the FoG episode by the algorithm. First, the average latency per participant was calculated by averaging the latencies of every individual FoG episode per participant. Hereafter, the average latency of every individual participant was averaged to determine the latency of the Cue2walk device.

### 2.5 Statistical Analysis

Statistical analysis was performed using GraphPad Prism 10.2.0 (GraphPad Software, San Diego, CA, USA). Differences in performance between the personally optimized setting and the standard setting of the Cue2walk device were assessed using paired *t*‐tests. Normality of the data were assessed using the Shapiro–Wilk test. If the data were not normally distributed, the Wilcoxon signed‐rank test was used to assess the differences in performance. A two‐tailed *p*‐value of < 0.05 was considered significant.

## 3 Results

### 3.1 Participant Characteristics

The characteristics of the 24 participants are summarized in Table 1.

**TABLE 1:** Participant characteristics.

Characteristic	Mean (standard deviation) or *n*
Age (years)	72.6 (6.6)
Disease duration (years)	8.8 (4.9)
FoG duration (years)	2.8 (2.2)
Sex (M/F)	16/8
MDS‐UPDRS part III	20.4 (9.2)^a^
Hoehn and Yahr stage	2.1 (0.5)^a^
MMSE	28.1 (1.8)^a^
Medication state (ON/OFF)	19/4^a^

Abbreviations: FoG, freezing of gait; MDS‐UPDRS, Movement Disorder Society Unified Parkinson's Disease Rating Scale; MMSE, Mini‐Mental State Examination.

^a^ Missing data in one participant.

### 3.2 FoG Video Annotation

**FIGURE 4:**
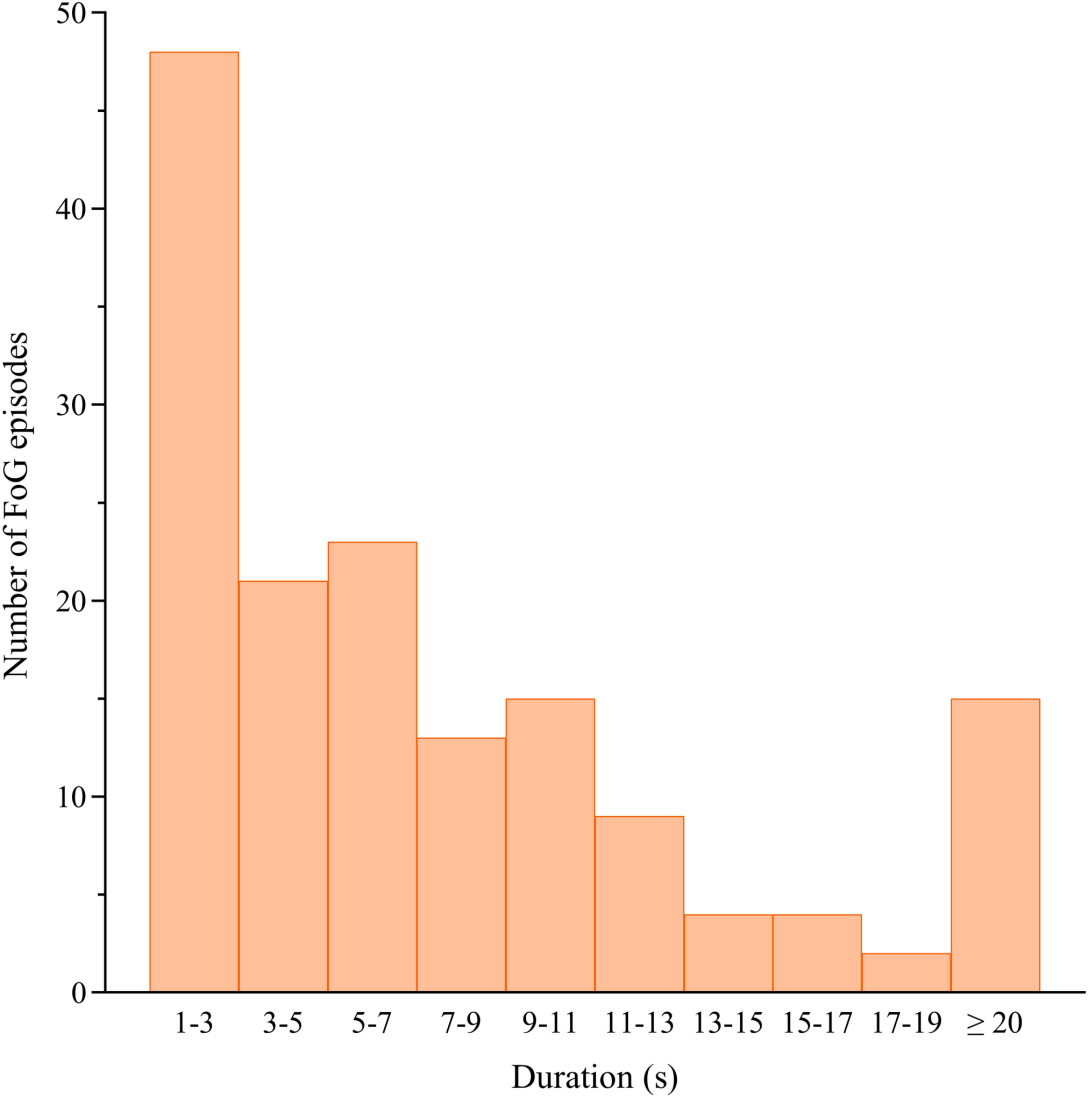
Histogram of the distribution of the duration of freezing of gait (FoG) episodes.

A total of 154 FoG episodes were annotated by the raters. The training set consisted of 79 FoG episodes, whereas the test set consisted of 75 FoG episodes. Mean duration of a FoG episode was 8.9 s (standard deviation [SD] 7.1 s) and 8.2 s (SD 4.9 s) in the training set and in the test set, respectively. Sixteen participants displayed FoG in both the training and test set, six participants did not display FoG during the entire measurement, and two participants did not display FoG in the training set, but did display FoG in the test set. The distribution of the duration of all FoG episodes is displayed in Figure 4.

### 3.3 Interrater Agreement

A total of 86 possible FoG episodes needed to be discussed because of discrepancy among the raters. It is important to note that for 13 possible FoG episodes, the discussion resulted in the possible episode being annotated as not a FoG episode. Cohen's Kappa was 0.66 (SD 0.22), indicating a substantial level of agreement between the raters. Prevalence index and bias index were −0.71 (SD 0.28) and −0.001 (SD 0.03), respectively. The high prevalence index suggests that the value for Cohen's Kappa is likely an underestimation of the level of agreement. The positive agreement was 0.71 (SD 0.18), whereas the negative agreement was 0.96 (SD 0.06), indicating, respectively, a substantial and almost perfect agreement between the raters.

### 3.4 Performance on Training Set

**FIGURE 5:**
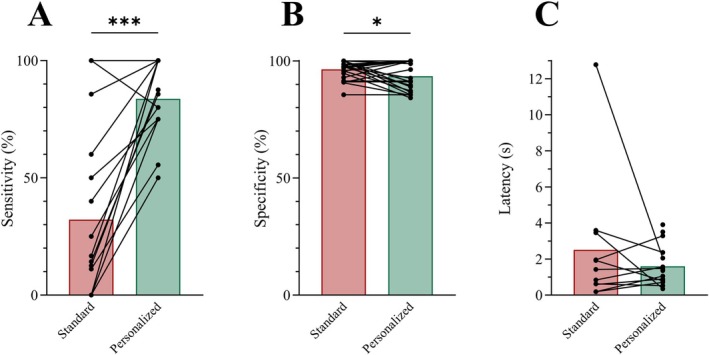
Performance to detect freezing of gait (FoG) of the Cue2walk device on the training set of the measurement. (A) Sensitivity for the 16 participants that displayed FoG in the training set and (B) specificity for all participants for the standard and personalized settings of the Cue2walk device for the training set. (C) Latency for the standard and personalized settings of the Cue2walk device for the training set. Note that in some participants, FoG episodes are only detected with the personalized and not the standard setting. *p < 0.05, **p < 0.01, ***p < 0.001.

In Figure 5A, the sensitivity for the standard and personally optimized settings of the Cue2walk device for the training set is displayed for the 16 participants that displayed FoG in the training set of the measurement. The sensitivity of the standard setting was 32.2% (SD 36.4%). Personal optimization of the settings of the algorithm significantly improved the sensitivity to 83.7% (SD 16.1%, *p* < 0.001).

Eight participants did not display FoG in the training set of the measurement, so sensitivity could not be calculated for these participants. Therefore, only specificity was determined for these participants. Specificity for the standard and personally optimized settings of the Cue2walk device for the training set for all 24 participants is displayed in Figure 5B. The specificity of the standard setting was 96.4% (SD 4.0%). Personal optimization of the settings resulted in a significantly lower specificity of 93.5% (SD 6.1%, *p* = 0.031).

In Figure 5C, the latency for the standard and personally optimized settings of the Cue2walk device for the training set is displayed. For the standard setting, the latency was 2.5 s (SD 3.6 s). For the personally optimized setting, the latency was 1.6 s (SD 1.1 s). The standard setting did not detect any FoG episodes in 5 of the 16 participants that displayed FoG in the training set of the measurement, whereas the personally optimized setting did detect FoG episodes in all of these 16 participants. Therefore, average latencies could be calculated for less participants when using the standard setting. Because paired *t*‐tests require the inclusion of participants with available average latency values for both settings, the statistical comparison was therefore restricted to this subset (*n* = 11). In this subset, personal optimization did not have an effect on the latency (2.5 s, SD 3.6 s [standard], vs. 1.4 s, SD 0.9 s [personalized], *p* > 0.05).

### 3.5 Performance on Test Set

**FIGURE 6:**
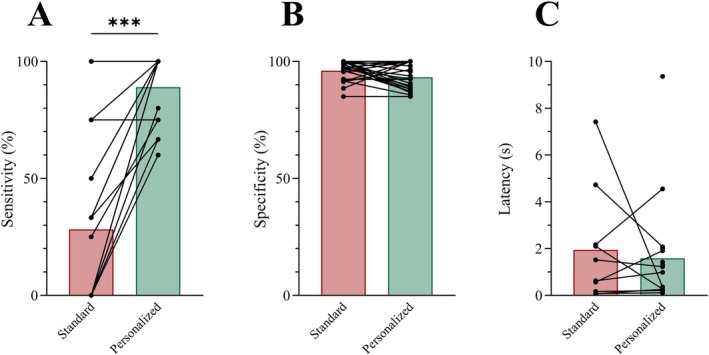
Performance to detect freezing of gait (FoG) of the Cue2walk device on the test set of the measurement. (A) Sensitivity for the 18 participants that displayed FoG in the test set and (B) specificity for all participants for the standard and personalized settings of the Cue2walk device for the test set. (C) Latency for the standard and personalized settings of the Cue2walk device for the test set. Note that in some participants, FoG episodes are only detected with the personalized and not the standard setting. *p < 0.05, **p < 0.01, ***p < 0.001.

In Figure 6A, the sensitivity for the standard and personally optimized settings of the Cue2walk device for the test set is displayed for the 18 participants that displayed FoG in the test set of the measurement. The sensitivity of the standard setting was 28.2% (SD 31.6%). Using the personally optimized setting, as determined on the training set, the sensitivity significantly improved to 89.1% (SD 14.8%, *p* < 0.001).

Six participants did not display FoG in the test set of the measurement, so sensitivity could not be calculated for these participants. Therefore, only specificity was determined for these participants. Specificity for the standard and personally optimized settings of the Cue2walk device for the test set for all 24 participants is displayed in Figure 6B. The specificity of the standard setting was 96.0% (SD 4.1%). Using the personally optimized setting, as determined on the training set, did not have an effect on the specificity (93.3%, SD 5.5%, *p* > 0.05).

In Figure 6C, the latency for the standard and personally optimized settings of the Cue2walk device for the test set is displayed. For the standard setting, the latency was 1.9 s (SD 2.4 s). For the personally optimized setting, as determined on the training set, the latency was 1.6 s (SD 2.2 s). In the subset of participants with available average latency values for both settings in the test set (*n* = 10), personal optimization did not have an effect on the latency (1.9 s, SD 2.4 s [standard], vs. 1.2 s, SD 1.4 s [personalized], *p* > 0.05).

### 3.6 Performance on Test Set of the Most and Least Sensitive Standard Setting

As described in the introduction, the Cue2walk device offers two more and two less sensitive options around the standard and personalized settings. These four settings are obtained by, respectively, lowering or raising the thresholds of the algorithm. Using the setting much more sensitive than the standard setting, sensitivity was higher (28.2%, SD 31.6% [standard], vs. 50.8%, SD 30.0% [much more sensitive], *p* = 0.001), and specificity was lower (96.0%, SD 4.1% [standard], vs. 86.5%, SD 13.9% [much more sensitive], *p* < 0.001) than the standard setting. In the subset of participants with available average latency values for both settings (*n* = 10), latency was shorter for the setting much more sensitive than the standard setting (1.9 s, SD 2.4 s [standard] vs. 0.7 s, SD 1.2 s [much more sensitive], *p* < 0.004).

Using the setting much less sensitive than the standard setting, sensitivity was similar (28.2%, SD 31.6% [standard], vs. 21.8%, SD 30.0% [much less sensitive], *p* > 0.05), and specificity was higher (96.0%, SD 4.1% [standard], vs. 98.4%, SD 2.8% [much less sensitive], *p* < 0.001) than the standard setting. In the subset of participants with available average latency values for both settings (*n* = 8), latency tended to be longer for the setting much less sensitive than the standard setting (1.2 s, SD 1.6 s [standard] vs. 1.6 s, SD 1.8 s [much less sensitive], *p* = 0.054).

## 4 Discussion

In this study, we investigated the performance of the Cue2walk CE class 1 medical device to detect FoG in terms of sensitivity, specificity, and latency. We tested the performance of the standard setting of the Cue2walk device, and we determined the effect of personal optimization of the detection algorithm on the performance to detect FoG. We found that for the standard setting, specificity was excellent and latency was good, but sensitivity was poor. Personal optimization of the settings led to a significantly improved sensitivity, while specificity and latency remained similar, resulting in a good performance to detect FoG of the Cue2walk device. Importantly, the performance to detect FoG remained stable on unseen data (i.e., the test set).

As described, the Cue2walk device is a single‐sensor device, which is placed just below the knee and uses a threshold algorithm as FoG detection method. Two previous studies investigated the performance to detect FoG of a single‐sensor device placed on the lateral side of the shank using a threshold algorithm as FoG detection method (Coste et al. 2014; Moore et al. 2013). Moore et al. (2013) achieved a sensitivity and specificity of 80%–90% and 60%–70%, respectively, depending on the chosen thresholds. Coste et al. (2014) achieved a sensitivity of 79.5%, but did not report on specificity. Differences in the detection algorithms and the methods used in the studies could explain the discrepancies in sensitivity and specificity with the present study. Moore et al. (2013) only used the Freeze Index for their FoG detection algorithm and determined sensitivity and specificity per timed up‐and‐go trial, whereas Coste et al. (2014) based their FoG detection algorithm on cadence and stride length and used a 10‐m‐straight walk with dual tasks. The Cue2walk device uses a combination of up to six algorithms, which likely provides a more robust detection performance than device set‐ups with only one algorithm. In addition to that, we used a walking trial in a real‐life setting as locomotor task, whereas sensitivity was determined based on every FoG episode, and specificity was determined per 1.5‐s bin in every trial. Taken together, although method of determining sensitivity and specificity and the walking tasks differed between the studies, the sensitivity of the Cue2walk device seems to be comparable or higher than similar device set‐ups, whereas specificity of the Cue2walk device seems to be higher.

Both of the studies investigating a similar device set‐up did not report on latency. Shorter latencies than the Cue2walk device are reported, but these devices often used multiple sensors (Ahn et al. 2017; Jovanov et al. 2009; Mazilu et al. 2015), limiting usability in daily life. Furthermore, if users of the Cue2walk device do want the cueing to start faster after the onset of a FoG episode, they have the option of increasing the sensitivity of the settings. In this way, the user can, indirectly, shorten the latency. This will however come at the cost of a lower specificity. Devices with multiple sensors often also report higher sensitivity and specificity (> 95%) (Elbatanouny et al. 2024; Zhang et al. 2024), which suggests a trade‐off between performance to detect FoG and usability in daily life. However, the Cue2walk device still achieves good performance to detect FoG with a single‐sensor device.

A limitation of this study is that, because we split the data from each measurement into a training set and a test set, the data sets were relatively small and contained a limited number of FoG episodes. A higher number of FoG episodes in the training set would have increased the input for the detection algorithm, so sensitivity could have been reduced in participants with a low number of FoG episodes in the training set. In addition to that, a higher number of FoG episodes in the test set would have increased the reliability of the performance to detect FoG, since one falsely missed FoG episode (false negative) can change sensitivity dramatically when the number of FoG episodes is low (e.g., 25% lower sensitivity in case of 4 FoG episodes). Furthermore, our approach of using the first 60% of each measurement for the training set generated a temporal bias of the data and resulted in the loss of participant data, since some participants did not display FoG in the training set. Another limitation is that, since we divided the measurements in relatively short bins of 1.5 s, the values we found for specificity could be high due to an inflated number of TN bins. Although we believe that our chosen bin duration is justified because an average users of the Cue2walk device takes approximately two steps in 1.5 s, future research should determine the effect of various bin durations on specificity. A fourth limitation is that one operator performed personal optimization of the settings, and that it was done via trial and error. With this approach, the optimization of the settings, and therefore the values found for sensitivity, specificity, and latency, will depend on the experience of the operator. Future studies should evaluate standardized or automated optimization procedures to improve reproducibility and reduce operator dependency. A last limitation is that no formal a priori power analysis was conducted. Because only one previous study was identified that determined the effect of personal optimization of their threshold‐based FoG detection algorithm on the performance to detect FoG (Bächlin et al. 2010), insufficient evidence was available to derive a reliable estimate of the expected effect size for sample size calculation. Consequently, the study may have been underpowered to detect small effects, and nonsignificant findings should therefore be interpreted with caution. The effect sizes observed in the current study may serve as a basis for future power analyses and sample size calculations in subsequent confirmatory investigations of this methodology.

As can be seen in Figures 5C and 6C, the standard setting detected a smaller fraction of the FoG episodes than the personally optimized setting. Because latency can only be determined for detected FoG episodes, the FoG episodes on which the average latency was determined differed between the settings. This was even more apparent in the settings less sensitive than the standard settings, in which an even smaller fraction of the FoG episodes was detected. Differences between the settings therefore need to be interpreted with caution.

A strength of this study is that the measurements were conducted in a real‐life setting (i.e., the house of the participant), closely reflecting the intended real‐world application of FoG detection devices. Another strength of this study is that we used a training set and a test set to determine the performance to detect FoG. However, the data sets are obtained from the same measurement for each participant and thus from the same day and the same context. Therefore, although we found that the performance to detect FoG remained stable on unseen test data, future studies should determine whether the performance is still maintained in other contexts, such as walking outside or in crowded spaces, and in multiple and prolonged sessions.

In conclusion, the Cue2walk smart cueing device, suitable for daily use in the own environment, showed good performance to detect FoG when the settings were personally optimized, and the performance was maintained on unseen test data. Future research needs to determine the clinical effectiveness and cost‐effectiveness of the device.

## Author Contributions

**Matthijs van der Laan:** conceptualization, data curation, formal analysis, methodology, visualization, writing – original draft, writing – review and editing. **Erwin E. H. van Wegen:** conceptualization, funding acquisition, methodology, supervision, writing – review and editing. **Jens A. N. Keijser:** conceptualization, data curation, methodology, software, writing – review and editing. **Sander A. L. M. Minnoye:** conceptualization, data curation, funding acquisition, methodology, software, writing – review and editing. **Rein van Zutven:** conceptualization, data curation, investigation, methodology. **Vincent de Groot:** methodology, writing – review and editing. **Marc B. Rietberg:** funding acquisition, methodology, supervision, writing – review and editing. **Jorik Nonnekes:** conceptualization, funding acquisition, methodology, supervision, writing – review and editing.

## Ethics Statement

The study was approved by the Medical Ethical Committee Arnhem‐Nijmegen (reference number 2023‐16413). Each participant gave written informed consent prior to entering the study.

## Conflicts of Interest

Jens A.N. Keijser, Sander A.L.M. Minnoye, and Rein van Zutven are employed at Cue2Walk International B.V. Jorik Nonnekes and Erwin E.H. van Wegen together form the medical advisory board of Cue2Walk International B.V., for which they receive no financial compensation. The other authors declare no conflicts of interest.

## Data Availability

The data that support the findings of this study are available from the corresponding author upon reasonable request.

## References

[ejn70589-bib-0001] Ahn, D. , H. Chung , H. W. Lee , et al. 2017. “Smart Gait‐Aid Glasses for Parkinson's Disease Patients.” IEEE Transactions on Biomedical Engineering 64: 2394–2402. 10.1109/TBME.2017.2655344. 28113199

[ejn70589-bib-0002] Bächlin, M. , M. Plotnik , D. Roggen , et al. 2010. “Wearable Assistant for Parkinson's Disease Patients With the Freezing of Gait Symptom.” IEEE Transactions on Information Technology in Biomedicine 14: 436–446. 10.1109/TITB.2009.2036165. 19906597

[ejn70589-bib-0003] Bloem, B. R. , J. A. Hausdorff , J. E. Visser , and N. Giladi . 2004. “Falls and Freezing of Gait in Parkinson's Disease: A Review of Two Interconnected, Episodic Phenomena.” Movement Disorders 19: 871–884. 10.1002/mds.20115. 15300651

[ejn70589-bib-0004] Byrt, T. , J. Bishop , and J. B. Carlin . 1993. “Bias, Prevalence and Kappa.” Journal of Clinical Epidemiology 46: 423–429. 10.1016/0895-4356(93)90018-V. 8501467

[ejn70589-bib-0005] Cicchetti, D. V. , and A. R. Feinstein . 1990. “High Agreement but Low Kappa: II. Resolving the Paradoxes.” Journal of Clinical Epidemiology 43: 551–558. 10.1016/0895-4356(90)90159-M. 2189948

[ejn70589-bib-0006] Cockx, H. , E. Klaver , M. Tjepkema‐Cloostermans , R. van Wezel , and J. Nonnekes . 2022. “The Gray Area of Freezing of Gait Annotation: A Guideline and Open‐Source Practical Tool.” Movement Disorders Clinical Practice 9: 1099–1104. 10.1002/mdc3.13556. 36339306 PMC9631855

[ejn70589-bib-0007] Coste, C. A. , B. Sijobert , R. Pissard‐Gibollet , M. Pasquier , B. Espiau , and C. Geny . 2014. “Detection of Freezing of Gait in Parkinson Disease: Preliminary Results.” Sensors (Basel) 14: 6819–6827. 10.3390/s140406819. 24740014 PMC4029660

[ejn70589-bib-0008] Cronin, P. , L. M. Collins , and A. M. Sullivan . 2024. “Impacts of Gait Freeze on Quality of Life in Parkinson's Disease, From the Perspectives of Patients and Their Carers.” Irish Journal of Medical Science 193: 2041–2050. 10.1007/s11845-024-03673-x. 38639839 PMC11294397

[ejn70589-bib-0009] Dvorani, A. , C. Wiesener , C. Salchow‐Hommen , et al. 2024. “On‐Demand Gait‐Synchronous Electrical Cueing in Parkinson's Disease Using Machine Learning and Edge Computing: A Pilot Study.” IEEE Open Journal of Engineering in Medicine and Biology 5: 306–315. 10.1109/OJEMB.2024.3390562. 38766539 PMC11100957

[ejn70589-bib-0010] Elbatanouny, H. , N. Kleanthous , H. Dahrouj , et al. 2024. “Insights Into Parkinson's Disease‐Related Freezing of Gait Detection and Prediction Approaches: A Meta Analysis.” Sensors (Basel) 24: 3959. 10.3390/s24123959. 38931743 PMC11207947

[ejn70589-bib-0011] Feinstein, A. R. , and D. V. Cicchetti . 1990. “High Agreement but Low Kappa: I. The Problems of Two Paradoxes.” Journal of Clinical Epidemiology 43: 543–549. 10.1016/0895-4356(90)90158-L. 2348207

[ejn70589-bib-0012] Folstein, M. F. , S. E. Folstein , and P. R. Mchugh . 1975. ““Mini‐Mental State”. A Practical Method for Grading the Cognitive State of Patients for the Clinician.” Journal of Psychiatric Research 12: 189–198. 10.1016/0022-3956(75)90026-6. 1202204

[ejn70589-bib-0013] Gámez‐Leyva, G. , and E. Cubo . 2024. “Freezing of Gait: Pharmacological and Surgical Options.” Current Opinion in Neurology 37: 394–399. 10.1097/WCO.0000000000001278. 38828625

[ejn70589-bib-0014] Gilat, M. , J. Nonnekes , S. A. Factor , et al. 2026. “An Updated Definition of Freezing of Gait.” Nature Reviews Neurology 22: 172–181. 10.1038/s41582-025-01179-3. 41513745

[ejn70589-bib-0015] Goetz, C. G. , B. C. Tilley , S. R. Shaftman , et al. 2008. “Movement Disorder Society‐Sponsored Revision of the Unified Parkinson's Disease Rating Scale (MDS‐UPDRS): Scale Presentation and Clinimetric Testing Results.” Movement Disorders 23: 2129–2170. 10.1002/mds.22340. 19025984

[ejn70589-bib-0016] Grimbergen, Y. A. , A. Schrag , G. Mazibrada , G. F. Borm , and B. R. Bloem . 2013. “Impact of Falls and Fear of Falling on Health‐Related Quality of Life in Patients With Parkinson's Disease.” Journal of Parkinson's Disease 3: 409–413. 10.3233/JPD-120113. 23948987

[ejn70589-bib-0017] Jovanov, E. , E. Wang , L. Verhagen , M. Fredrickson , and R. Fratangelo . 2009. “deFOG‐A Real Time System for Detection and Unfreezing of Gait of Parkinson's Patients.” 2009 Annual International Conference of the IEEE Engineering in Medicine and Biology Society 2009: 5151–5154. 10.1109/IEMBS.2009.5334257. 19964859

[ejn70589-bib-0018] Landis, J. R. , and G. G. Koch . 1977. “The Measurement of Observer Agreement for Categorical Data.” Biometrics 33: 159–174. 10.2307/2529310. 843571

[ejn70589-bib-0019] Latt, M. D. , S. R. Lord , J. G. Morris , and V. S. Fung . 2009. “Clinical and Physiological Assessments for Elucidating Falls Risk in Parkinson's Disease.” Movement Disorders: Official Journal of the Movement Disorder Society 24: 1280–1289. 10.1002/mds.22561. 19425059

[ejn70589-bib-0020] Lewis, S. J. G. , and J. M. Shine . 2016. “The Next Step: A Common Neural Mechanism for Freezing of Gait.” Neuroscientist 22: 72–82. 10.1177/1073858414559101. 25398230

[ejn70589-bib-0021] Mazilu, S. , U. Blanke , M. Dorfman , et al. 2015. “A Wearable Assistant for Gait Training for Parkinson's Disease With Freezing of Gait in Out‐of‐the‐Lab Environments.” ACM Transactions on Interactive Intelligent Systems 5: 1–31. 10.1145/2701431.

[ejn70589-bib-0022] Minnoye, A. L. M. , F. Waardenburg , and M. van der Ent . 2023. Cueing Device Algorithm (WO2023161359A1). World Intellectual Property Organization. https://worldwide.espacenet.com/patent/search/family/085278025/publication/WO2023161359A1?q=pn%3DWO2023161359A1.

[ejn70589-bib-0023] Molina, R. , C. J. Hass , S. Cernera , et al. 2021. “Closed‐Loop Deep Brain Stimulation to Treat Medication‐Refractory Freezing of Gait in Parkinson's Disease.” Frontiers in Human Neuroscience 15: 633655. 10.3389/fnhum.2021.633655. 33732122 PMC7959768

[ejn70589-bib-0024] Moore, O. , C. Peretz , and N. Giladi . 2007. “Freezing of Gait Affects Quality of Life of Peoples With Parkinson's Disease Beyond Its Relationships With Mobility and Gait.” Movement Disorders 22: 2192–2195. 10.1002/mds.21659. 17712856

[ejn70589-bib-0025] Moore, S. T. , H. G. MacDougall , and W. G. Ondo . 2008. “Ambulatory Monitoring of Freezing of Gait in Parkinson's Disease.” Journal of Neuroscience Methods 167: 340–348. 10.1016/j.jneumeth.2007.08.023. 17928063

[ejn70589-bib-0026] Moore, S. T. , D. A. Yungher , T. R. Morris , et al. 2013. “Autonomous Identification of Freezing of Gait in Parkinson's Disease From Lower‐Body Segmental Accelerometry.” Journal of Neuroengineering and Rehabilitation 10, no. 19: 19. 10.1186/1743-0003-10-19. 23405951 PMC3598888

[ejn70589-bib-0027] Nieuwboer, A. , G. Kwakkel , L. Rochester , et al. 2007. “Cueing Training in the Home Improves Gait‐Related Mobility in Parkinson's Disease: The RESCUE Trial.” Journal of Neurology, Neurosurgery and Psychiatry 78: 134–140. 10.1136/jnnp.200X.097923. 17229744 PMC2077658

[ejn70589-bib-0028] Nieuwboer, A. , L. Rochester , T. Herman , et al. 2009. “Reliability of the New Freezing of Gait Questionnaire: Agreement Between Patients With Parkinson's Disease and Their Carers.” Gait & Posture 30: 459–463. 10.1016/j.gaitpost.2009.07.108. 19660949

[ejn70589-bib-0029] Nonnekes, J. , E. Ruzicka , A. Nieuwboer , M. Hallett , A. Fasano , and B. R. Bloem . 2019. “Compensation Strategies for Gait Impairments in Parkinson Disease: A Review.” JAMA Neurology 76: 718–725. 10.1001/jamaneurol.2019.0033. 30907948

[ejn70589-bib-0030] Poewe, W. 2008. “Non‐Motor Symptoms in Parkinson's Disease.” European Journal of Neurology 15: 14–20. 10.1111/j.1468-1331.2008.02056.x. 18353132

[ejn70589-bib-0031] Porciuncula, F. , J. T. Cavanaugh , J. Zajac , et al. 2025. “Amplifying Walking Activity in Parkinson's Disease Through Autonomous Music‐Based Rhythmic Auditory Stimulation: Randomized Controlled Trial.” Npj Parkinson's Disease 11: 100. 10.1038/s41531-025-00952-x. PMC12041193 40301366

[ejn70589-bib-0032] Rider, J. V. , K. L. C. Manalang , and J. K. Longhurst . 2025. “Freezing of Gait Is Associated With Daily Activity Limitations Among Individuals With Parkinson's Disease and Mild Cognitive Impairment.” Occupational Therapy in Health Care 39: 361–375. 10.1080/07380577.2024.2314181. 38343304

[ejn70589-bib-0033] Sim, J. , and C. C. Wright . 2005. “The Kappa Statistic in Reliability Studies: Use, Interpretation, and Sample Size Requirements.” Physical Therapy 85: 257–268. 10.1093/ptj/85.3.257. 15733050

[ejn70589-bib-0034] Tosserams, A. , A. Fasano , M. Gilat , et al. 2025. “Management of Freezing of Gait—Mechanism‐Based Practical Recommendations.” Nature Reviews Neurology 21: 327–344. 10.1038/s41582-025-01079-6. 40169855

[ejn70589-bib-0035] van der Laan, M. , M. B. Rietberg , M. van der Ent , et al. 2025. “User Experiences of the Cue2walk Smart Cueing Device for Freezing of Gait in People With Parkinson's Disease.” Sensors (Basel) 25: 4702. 10.3390/s25154702. 40807870 PMC12349309

[ejn70589-bib-0037] Zhang, W. S. , C. Gao , Y. Y. Tan , and S. D. Chen . 2021. “Prevalence of Freezing of Gait in Parkinson's Disease: A Systematic Review and Meta‐Analysis.” Journal of Neurology 268: 4138–4150. 10.1007/s00415-021-10685-5. 34236501

[ejn70589-bib-0036] Zhang, W. , H. Sun , D. B. Huang , et al. 2024. “Detection and Prediction of Freezing of Gait With Wearable Sensors in Parkinson's Disease.” Neurological Sciences 45: 431–453. 10.1007/s10072-023-07017-y. 37843692

[ejn70589-bib-0038] Zoetewei, D. , T. Herman , P. Ginis , et al. 2024. “On‐Demand Cueing for Freezing of Gait in Parkinson's Disease: A Randomized Controlled Trial.” Movement Disorders 39: 876–886. 10.1002/mds.29762. 38486430

